# pH-responsive stealth micelles composed of cholesterol-modified PLA as a nano-carrier for controlled drug release

**DOI:** 10.1007/s40204-014-0022-y

**Published:** 2014-04-03

**Authors:** Massoumeh Bagheri, Elham Bigdeli, Zhaleh Pourmoazzen

**Affiliations:** Chemistry Department, Science Faculty, Azarbaijan Shahid Madani University, P.O. Box: 53714-161, 5375171379 Tabriz, Iran

**Keywords:** l-lactide, Cholesterol, 2-(Dimethylamino)ethyl methacrylate, Amphiphilic brush copolymer, Macromonomer, pH-responsive micelles

## Abstract

Present research is a preliminary report on the novel pH-responsive micelles based on an amphiphilic brush copolymer P(PEGMA)-*b*-P(DMAEMA-*co*-CPLAMA) used as the promising drug carrier. The copolymer was synthesized using cholesteryl poly(l-lactic acid) methacrylate (CPLAMA), poly(ethylene glycol) monomethyl ether methacrylate (PEGMA) and 2-(dimethylamino)ethyl methacrylate (DMAEMA) with appropriate hydrophobic/hydrophilic ratios via atom transfer radical polymerization. The copolymer compositions were determined by ^1^H NMR. The synthesized copolymer self-assembled into nano-scale micelles capable of encapsulating hydrophobic model drug naproxen in their hydrophobic cores in aqueous solutions. pH sensitivity and self-assembly behaviors of copolymer were studied by UV–vis transmittance, fluorescence spectroscopy, transmission electron microscopy (TEM) and dynamic light scattering. The results showed that the copolymer had high pH responsivity with a phase transition pH around pH 6.2. The critical micelle concentrations at pH 6.5 were found about 2.4 mg L^−1^. The stable and small micelles were obtained at pH 5.5–6.5. Upon increasing pH higher than 7, the single micelles further assembled into the micellar aggregates. TEM images of copolymer micelles showed that the micelles are spherical in shape with the mean diameter of 152 nm at pH 6.2. In vitro release study of naproxen-loaded micelles with about 44 % loading efficiency and 8 % loading capacity was performed using dialysis method in phosphate-buffered solution at 37 °C. Release study implied that the proposed brush copolymer could produce stable nano-carriers with controllable drug release at the target sites (pH 5.5–7).

## Introduction

In recent decades, there has been increased awareness of the necessity for the development of drug delivery systems to improve the properties of therapeutic compounds, increasing its effectiveness, and decreasing its harmful side effects (Allen and Cullis 2004). To achieve effective targeted delivery and satisfactory therapeutic applications, various natural and synthetic polymers have been used for the delivery of therapeutic agents such as drugs and genes (Bagheri and Pourmirzaei 2013). Among synthetic polymers, amphiphilic polymers are self-assembled in aqueous solution to minimize contact between water and hydrophobic blocks, and can contain hydrophobic drugs in the inner hydrophobic core (Geun-woo et al. 2009). Drug delivery systems capable of releasing their payload in response to stimuli have drowned much attention in recent years, whether to target tissues, to reach specific intracellular locations, or to promote drug release. Stimuli-responsive polymers show a sharp change in properties upon a small or modest changes in environmental condition, e.g., temperature, light, salt concentration or pH (Schmaljohann 2006).

Polymeric micelles are nano-scopic constructs that possess core/shell architecture. They are obtained from the self-assembly of amphiphilic block copolymers in an isotropic aqueous solution above the critical micelle concentration (CMC). The core, consisting of the hydrophobic domain, acts as a reservoir and protects the drug payload whereas the hydrophilic shell mainly confers aqueous solubility and steric stability to the ensemble (Yang et al. 2011).

The design and preparation of pH-sensitive micelles are an exciting field of research that seeks to exploit the attractive properties of polymeric micelles to improve the selective delivery of therapeutic molecules using physiological triggers. Researchers attempting to exploit pH-sensitive micellar systems for drug delivery applications have generally focused on systems having transitions in the physiologically accessible pH ranges (Allen and Cullis 2004). Ionisable polymers with a p*K*a value between 3 and 10 are candidates for pH-responsive systems (Li et al. 2011). Different organs, tissues and cellular compartments may have large differences in pH, which make the pH a suitable stimulus. In addition, it is proposed that micelles are taken up by cells via an endocytosis process (Luo et al. 2002). While the endocytic pathway begins near the physiological pH of 7.4, it drops to a lower pH (5.5–6.0) in endosomes and approaches pH 5.0 in lysosomes. Therefore, polymeric micelles responsive to the pH gradients can be designed to release their payload selectively in tumor tissue or within tumor cells (Felber et al. 2011). One of the major challenges has been the relatively narrow pH range in which the micellar carrier must both retain the drug over prolonged periods and then release it relatively rapidly. This challenge has been met by many different approaches including incorporation of titratable groups into the copolymer backbone such that the solubility of the polymer is altered by protonation or deprotonation events. Poly(2-(dimethylamino)ethyl methacrylate) (PDMAEMA) is among the hydrophilic polymers, with pH and temperature sensitivities and excellent biocompatibility Zhou et al. (2010). PDMAEMA is partially positively charged in water. Micelles with PDMAEMA chains have a positively charged hydrophilic surface, which may facilitate the cellular uptake by adsorption mediated endocytosis (Miller et al. 1998).

The amphiphilic pH-sensitive copolymers consisting of poly(ε-caprolactone) and PDMAEMA segments arranged in diblock (Bougard et al. 2007), triblock (Zhu et al. 2005) graft (Jhurry and Motala-Timol 2007; Nottelet et al. 2008), brush (Xu et al. 2004) and miktoarm star (Liu et al. 2007) architectures have been synthesized as drug carriers for targeted drug delivery (Xu et al. 2004; Zhou et al. 2010). Armes and coworkers (1999) have prepared a series of block copolymers having PDMAEMA, poly [2-(*N*-morpholino)ethyl methacrylate] (De Paz Bánez et al. 2001) and poly(ethylene glycol) (PEG) groups (Liu et al. 2001).

These copolymers exhibit pH-dependent micellization owing to their tertiary amine groups, with the behavior of each block copolymer depending on the hydrophobicity and p*K*a of the specific amine block involved. The transitional pH is typically in the range of 6–7 for these systems. Recently, Zhang et al. (Zhang et al. 2010) reported the preparation of block-brush copolymer of [PEG-*block*-P(NIPAM-*graft*-PDMAEMA)] by the combination of ATRP and click chemistry as dually thermo- and pH-responsive copolymer.

On the other hand, poly(l-lactic acid) (PLA) and its copolymers have attracted much attention as materials for controlled drug-releasing applications due to the excellent biodegradability, biocompatibility and low toxicity (Bagheri and Motirasoul 2012). In our previous works, amphiphilic block copolymers containing the hydrophobic cholesteryl-modified poly l-lactic acid (CPLA) block and different hydrophilic blocks, such as poly(glycidyl methacrylate) (PGMA) and PEG have been synthesized (Bagheri and Motirasoul 2013; Bagheri et al. 2013).

The purpose of this work was to synthesize and study the new polymeric stealth micelle systems that were designed based on the concept of the pH-responsive nano-carriers. In the previous work, we synthesized biodegradable and biocompatible brush poly[cholesteryl-(l-lactic acid)_*n*_ methacrylate]-co-Poly[poly(ethylene glycol)monomethyl ether methacrylate], P(CPLAMA)-co-P(PEGMA), copolymers as a stealth micellar nano-carriers (Bagheri and Bigdeli 2013). This paper is a kind of preliminary report of the synthesis and characterization of new amphiphilic pH-sensitive brush copolymer P(PEGMA)-*b*-P(DMAEMA-*co*-CPLAMA) via ATRP of corresponding monomers (Rajesh and James 2009). We have largely focused on the self-assembly of copolymer into micelle aggregates with different structure upon the pH-induced increase in hydrophobicity/hydrophilicity of the polymer structure. Moreover, initial studies on in vitro release of naproxen as a hydrophobic model drug have been described.

## Experimental

### Materials

**Scheme 1:**
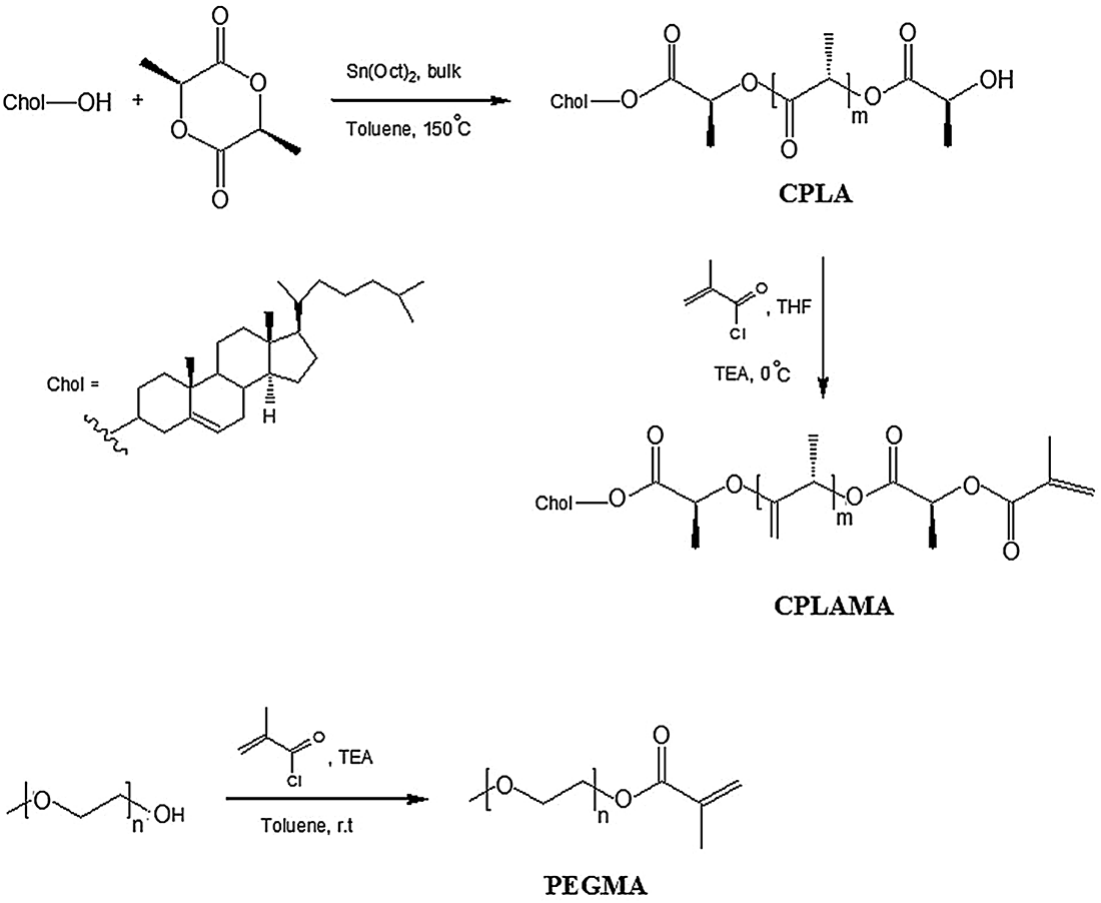
Synthetic routes for CPLAMA and PEGMA

**Scheme 2:**
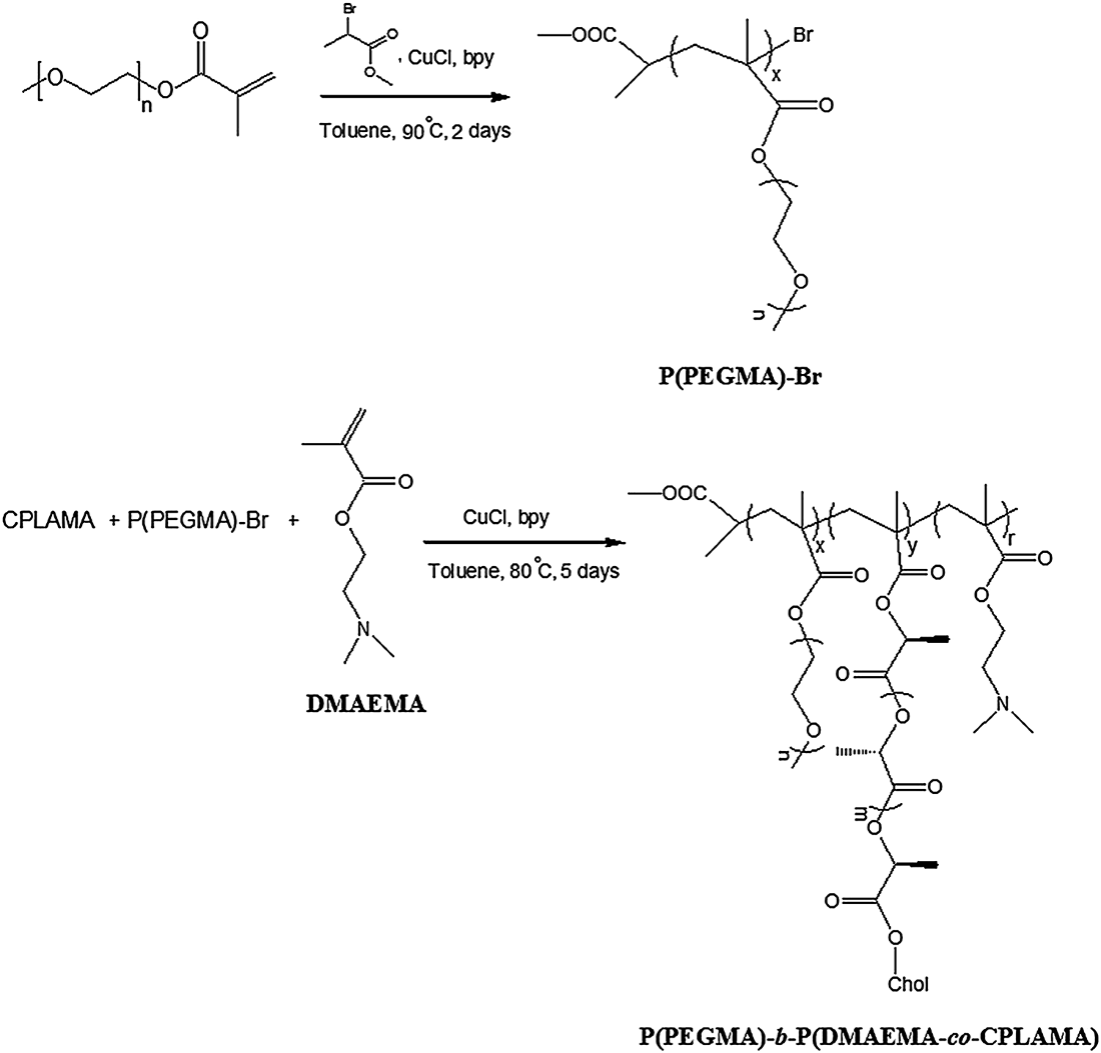
Synthetic routes for P(PEGMA)-Br and P(PEGMA)-*b*-P(DMAEMA-*co*-CPLAMA)

l-lactide, tin (II) bis (2-ethylhexanoate) (Sn(Oct)_2_), and triethyl amine (TEA) were obtained from Alfa Aesar. Methacryloyl chloride (96 %), aluminum oxide (Al_2_O_3_), copper (I) chloride (CuCl), 2, 2^′^-bipyridine (bpy), methyl-2-bromopropionate (MBP), cholesterol and naproxen were purchased from Merck (Germany). 2-(Dimethylamino)ethyl methacrylate (DMAEMA) and Poly (ethylene glycol) (mPEG) (*M*_n_ = 5,000 g mol^−1^ according to the manufacturer) were purchased from Aldrich (USA). Dialysis membrane (MWCO = 10,000) was purchased from Sigma-Aldrich (USA). DMA was passed through basic alumina columns, then vacuum-distilled from CaH_2_, and stored at −20 °C prior to use. PEG dried under vacuum for about 24 h before use. TEA was refluxed for 12 h in the presence of CaH_2_ and distilled in vacuo. CuCl was purified by precipitation from glacial acetic acid to remove Cu^2+^, filtered and washed with ethanol, and then dried. Cholesterol was purified by recrystallization in methanol. Tetrahydrofuran (THF) and toluene were dried by refluxing over sodium. The procedures of the synthesis of CPLAMA and PEGMA were detailed in our previous paper (Scheme 1) (Bagheri and Bigdeli 2013). The copolymers were synthesized according to the route outlined in Scheme 2. Further details are given below.

### Synthetic procedures

#### Bromo-ended poly[poly(ethylene glycol) monomethyl ether methacrylate] macroinitiator (P(PEGMA)-Br)

P(PEGMA)-Br macroinitiator was synthesized by ATRP using the MBP as the initiator in toluene at 60 °C with the ratio of reagents [PEGMA]: [MBP]:[CuCl]:[bpy] = 3:1:1:1. PEGMA (*M*_n_ = 5.07 × 10^3^, 1 g, 0.197 mmol), bpy (10.15 mg, 0.065 mmol), and CuCl (6.5 mg, 0.065 mmol) charged to a Schlenk tube. The mixture was degassed by vacuum/nitrogen purging for three cycles. Degassed toluene (1 mL) was added by a syringe. The mixture was heated in water bath at 40 °C for 2 min, and a clear solution was formed. This solution was degassed again for 5 min under stirring. The initiator MBP (7.32 μL, 0.065 mmol) was added by a syringe, and then the tube was immersed in water bath at 60 °C. After 2 days, the reaction mixture was dissolved in toluene and passed through an aluminum oxide column to remove the copper catalyst and then precipitated in methanol to remove the unreacted macromonomer. The final product was isolated and dried under vacuum for 48 h. The yield was 0.69 g (70 %). FT-IR (KBr) *υ*: 2,888 (C–H, stretch), 1,717 (C=O, stretch), 1,467 (CH_2_, bend), 1,360 (CH_3_, bend), 1,149 and 1,111 (C–O, stretch) cm^−1^. ^1^H NMR (400 MHz, CDCl_3_) *δ*: 4.30 (t, 2H, –C*H*_2_OCO– from PEG), 3.75 and 3.57 (br, nH, –*CH*_*2*_ *CH*_*2*_O– from PEG), 3.49 (m, 3H, –COO*CH*_*3*_ from MBP), 3.47 (s, 3H, –O*CH*_*3*_ from PEG), 1.7 (m, 1H, H_3_CC*H*COOCH_3_ from MBP), 1.56 (d, 3H, C*H*_*3*_ from MBP), 1.27 (s, –*CH*_*3*_ from backbone) ppm.

#### Poly[poly(ethylene glycol)monomethyl ether methacrylate]-b-[2-(dimethylamino)ethyl methacrylate-co-cholesteryl-(l-lactic acid)_*n*_ methacrylate], (P(PEGMA)-b-P(DMAEMA-co-CPLAMA))

The synthesis of copolymer (P(PEGMA)-*b*-P(DMAEMA-*co*-CPLAMA)), which has a 1:1 molar ratio of DMAEMA to CPLAMA, was synthesized by conducting “grafting-through” ATRP using the PEGMA-Br as the macroinitiator in toluene at 80 °C with the ratio of reagents [CPLAMA + DMAEMA]:[PEGMA-Br]:[CuCl]:[bpy] = 10:1:1:1. PEGMA-Br (*M*_n NMR_ = 11.237 × 10^3^, 0.45 g, 0.04 mmol), CPLAMA (*M*_n_ = 3.77 × 10^3^, 0.73 g, 0.2 mmol), bpy (6.24 mg, 0.04 mmol), and CuCl (3.95 mg, 0.04 mmol) were charged to a Schlenk tube. After three freeze-pump thaw cycles, Degassed toluene (1 mL) was added by a syringe. The mixture was heated in water bath at 40 °C for 2 min, and a clear solution was formed. This solution was degassed again for 5 min under stirring. DMAEMA (33.7 μL, 0.2 mmol) was added by a syringe, and then the tube was immersed in water bath at 80 °C. After 5 days, the reaction mixture was dissolved in toluene and passed through an aluminum oxide column to remove the copper catalyst and then precipitated in *n*-hexane. The final product was isolated and dried under vacuum for 48 h. The yield was 0.38 g (60 %). FT-IR (KBr) *υ*: 2,948 and 2,888 (C–H, stretch), 1,758 and 1,717 (C=O, stretch), 1,467 (CH_2_, bend), 1,360 (CH_3_, bend), 1,280 (C–N, stretch), 1,194 and 1,111 (C–O, stretch), 842 (=C–H, (OOP) bend) cm^−1^. ^1^H NMR (400 MHz, CDCl_3_) *δ*: 5.38 (m, 1H, =C*H* from cholesterol), 5.18 (q, nH, –(O(C = O)C*H*(CH_3_))_*n*_–), 4.61 (m, 1H, –C*H* from cholesterol), 4.30 (t, 2H, –C*H*_2_CH_2_N(CH_3_)_2_ from DMAEMA), 3.74–3.54 (br, nH, –*CH*_*2*_ *CH*_*2*_O– from PEG), 3.39 (s, 3H, –COOCH3), 3.37 (s, 3H, –O*CH*_*3*_ from PEG), 2.75 (t, 2H, –CH_2_C*H*_2_N(NH_3_)_2_ from DMAEMA), 2.42 (br, 6H, –N(C*H*_3_)_2_ from DMAEMA), 2.08 (m, 3H, –COOC*H*_3_), 1.96 (m, H_3_CC*H*COOCH_3_), 1.76 (–CH_3_ from MBP), 1.62 (q, –*CH*_*2*_ methacrylate backbone), 1.56 (d, 3nH, –(O(C=O)CH(*CH*_*3*_))_*n*_–), 1.46 (s, 3H, –C*H*_*3*_ from cholesterol), 1.34 (s, –*CH*_*3*_ methacrylate backbone), 1.01 (d, 3H, C*H*_*3*_ from cholesterol), 0.92 (d, 3H, C*H*_*3*_ from cholesterol), 0.84 (d, 2 × 3H, C*H*_*3*_ from cholesterol), 0.7–2.37 (b, 28H, H of C*H*_*2*_ and C*H* from cholesterol), 0.67 (s, 3H, *C*-*18 H* from cholesterol), ppm.

#### Preparation of polymeric micelles

The blank polymeric micelles were prepared using a co-solvent evaporation method. In brief, P(PEGMA)-*b*-P(DMAEMA-*co*-CPLAMA) copolymer (2 mg) was dissolved in 1 mL of THF in a 10-mL flask, and then the solution of the polymer was added dropwise into 6 mL of buffer solutions at desired pH under high-speed stirring. Finally, the mixed solution was transferred to a beaker and slowly stirred for 24 h at room temperature to facilitate the removal of THF.

#### Drug encapsulation

Low aqueous solubility drug naproxen was used as a model drug for investigating the loading and release properties of drug in the polymer carrier. The naproxen-loaded polymeric micelles were prepared as follows. The P(PEGMA)-*b*-P(DMAEMA-*co*-CPLAMA) (10 mg) and naproxen (2 mg) were dissolved in 1 mL of THF, and the solution was added into 6 mL buffer solution and stirred for 24 h. After the THF was removed by evaporation, micelle-dispersed solution was obtained. The obtained suspension was centrifuged at 4,000 rpm for 10 min, and then the supernatant containing naproxen-loaded micelles was obtained. Drug-loaded polymeric micelles were lyophilized. The precipitate containing unloaded drug was dissolved in 50 % THF solution, and its amount was analyzed by UV–visible spectrophotometry at 330 nm.

Drug loading efficiency and drug loading capacity (Table 2) were calculated as follows:

$$\text{Loading capacity}\;\left(\%\right)=\left(A-B\right)/C\times 100$$

$$\text{Loading efficiency}\;\left(\%\right)=\left(A-B\right)/A\times 100,$$

where *A* is the total weight of naproxen used, *B* is the weight of unloaded naproxen in the precipitate after centrifugation and *C* is the weight of copolymer.

#### In vitro drug release test

In vitro release of naproxen from the micelle solution was determined using the dialysis membrane diffusion technique. Three milliliters of drug-loaded micelle solution was transferred into a dialysis tube (MWCO = 10,000) and immersed into 30 mL of release media [pH 3–12 phosphate-buffered solutions (PBS)] at 37 °C and stirred at 250 rpm. At predetermined intervals, 3 mL of the medium was taken and 3 mL of fresh PBS was added after each removal. Concentration of the drug released was determined using a UV–visible spectrophotometer at 330 nm, and all experiments were carried out in triplicate. The standard aqueous solutions were prepared at concentrations ranging from 0.001 to 0.01 g L^−1^. The correlation coefficient (*R* ^2^) value was at least 0.996. The release percentage of naproxen was calculated from the following equation:

$$\%\;\text{Release}=\left(W_{t}\right)/\left(W_{\text{total}}\right)\times \;100,$$

where *W*_*t*_ is the weight of released naproxen at time *t* and *W*_total_ is the total absorbed naproxen in the polymeric micelle structure. *W*_total_ was calculated by the free drug amount, i.e., the total drug amount used in this work; here it is 2 mg (*A*) minus the amount of unloaded drug (*B*).

### Characterization

Spectroscopic characterization is utilized by the following instrumentations: Melting points were recorded with an electrothermal (Rochford, UK) 9100 apparatus. FT-IR Spectra were recorded on a Brucker PS-15 spectrometer (Bruker Optics, Ettlingen, Germany). ^1^H NMR Spectra were taken on a 400 and 300 MHz Brucker SP-400 Advance spectrometer (Bruker Biospin, Rheinstetten, Germany) using chloroform as solvent with tetramethylsilane as the internal standard. The pH measurements were carried out with a Metrohm (Herisau, Switzerland) 774 pH meter equipped with glass and Ag/AgCl reference electrodes calibrated with standard HCl and acetate buffer at an ionic strength of 0.10 M (NaCl). Fluorescence spectra were recorded on a LS50B luminescence spectrometer (Perkin-Elmer, USA) at room temperature. The morphology of the self-assembly micelles in aqueous solution (2.00 mg mL^−1^) was observed by transmission electron microscopy (TEM) (PHILIPS SM10 TEM, The Netherlands, and EPSON HP8300 Photo flat-bed scanner operated at an accelerating voltage of 150 keV). Dynamic light scattering (DLS) measurements were performed on the Zetasizer instrument ZEN3600 (Malvern, UK MAL1001767) with a He–Ne laser beam at 511 nm at 25 °C. Samples were filtered with a 0.2 μm filter of mixed cellulose acetate to remove any interfering dust particles. Optical density (OD) measurements were done at 500 nm on a UV–VIS recording spectrophotometer (ShimadzuUV160 A, Japan).

## Results and discussion

### Synthesis and structural characterization

The synthesized amphiphilic biodegradable and biocompatible brush copolymer P(CPLAMA)-*co*-P(PEGMA) through ATRP of PEGMA and CPCLAMA has been reported earlier for drug delivery applications (Bagheri and Bigdeli 2013). The aim of the present work was to modify this amphiphilic brush copolymer by introducing DMAEMA monomer as a new class of pH-responsive material to preparation of nano-carrier for controlled drug release.

The synthesis and characterization of CPLAMA and PEGMA have been previously reported (Bagheri and Bigdeli 2013). The synthetic route of macromonomers, CPLAMA and PEGMA, is presented in Scheme 1.

In the second step, PEGMA was subsequently employed as a macromonomer to induce its ATRP. According to our previous study, the high concentration of the catalyst and initiator was found to be necessary for high yield of macromonomer polymerization. The synthetic routes for macroinitiator P(PEGMA)-Br and the amphiphilic P(PEGMA)-*b*-P(DMAEMA-*co*-CPLAMA) copolymer are shown in Scheme 2.

**Fig. 1:**
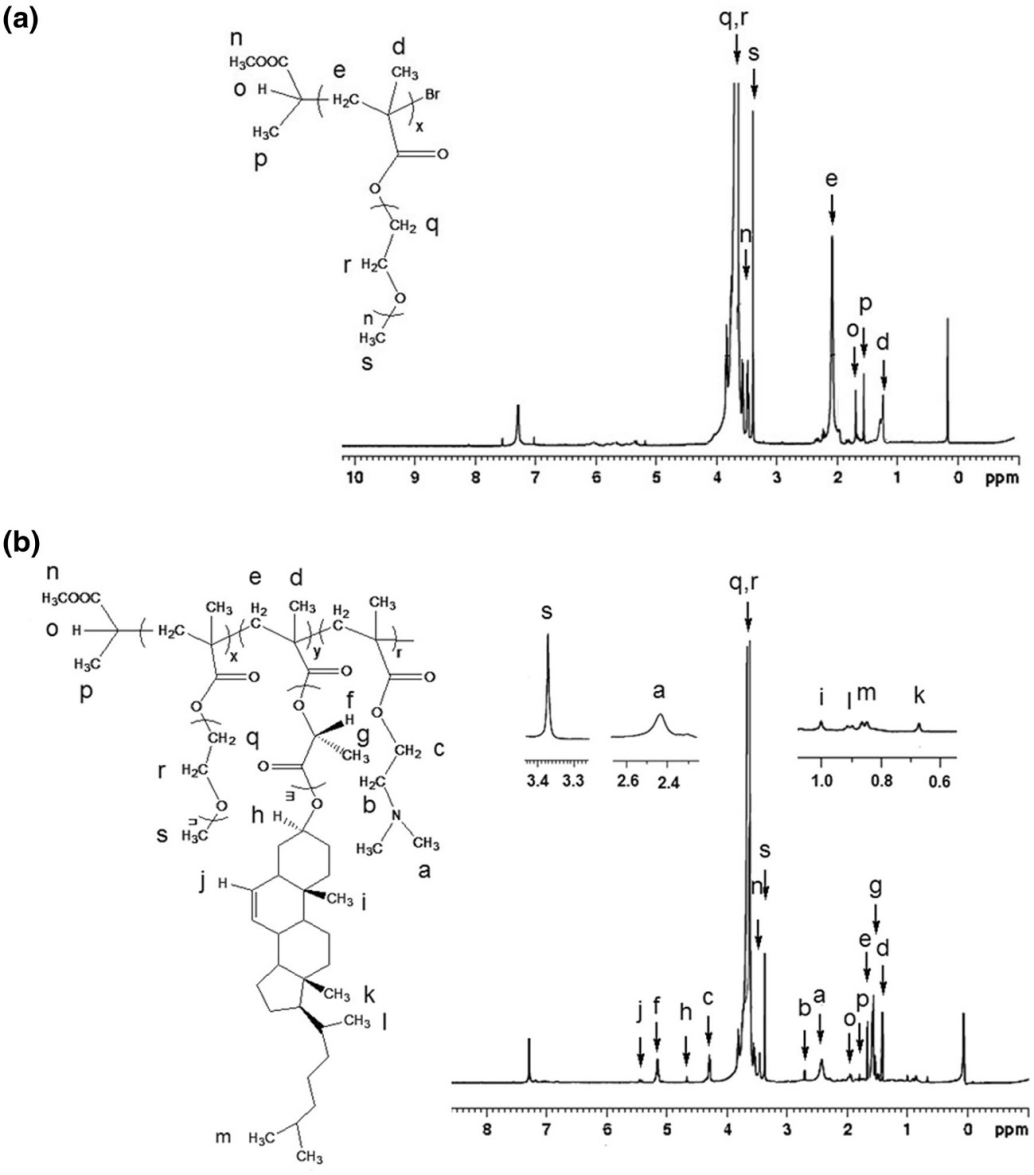
^1^H NMR spectra of P(PEGMA)-Br, and P(PEGMA)-*b*-P(DMAEMA-*co*-CPLAMA)

P(PEGMA) was obtained by ATRP of PEGMA monomer using MBP as small molecular initiator, CuCl/bpy as the catalyst system at 60 °C in 3 mL toluene. The components of molar ratio were designed as [PEGMA]:[CuCl]:[bpy]:[MBP] = 3:1:1:1 (Table 1). ^1^H NMR spectrum of P(PEGMA)-Br is shown in Fig. 1. In addition to the dominant PEG signals at 3.47 and 3.57–3.75 ppm, the characteristic signals (*n*, *p*) of two methyl groups of initiator moiety could be pointed out clearly at 3.49 and 1.56 ppm, respectively, which clarified the MBP-initiated ATRP of PEGMA. Quartet signal (*o*) at 1.7 ppm represented the initiator methine protons of the MBP, while the singlet signals (*d*) at 1.27 ppm correspond to the methyl protons of the PEGMA main-chain (Fig. 1).

**Table 1:** Characterization of the synthesized P(PEGMA)-Br and P(PEGMA)-*b*-P(DMAEMA-*co*-CPLAMA)

Sample	Molar ratio of initiator to monomer (s) (%)		Molar ratio of CPLA: DMAEMA (%)		GPC result	
	Feed ratio	Calculated	Feed ratio	Calculated	*M*_n_ (g mol^−1^)	*M*_w_/*M*_n_
P(PEGMA)-Br	1:3	1:2.25	–	–	–	–
P(PEGMA)-*b*-P(DMAEMA-*co*-CPLAMA	1:10	1:9.46	1:1	1:1.49	32,971	1.86

The prepared macroinitiator was used to polymerize the CPLAMA and DMAEMA subsequently. ATRP of CPLAMA and DMAEMA using P(PEGMA)-Br initiator was carried out at 80 °C in 3 mL toluene with CuCl/bpy as the catalyst system. ^1^H NMR spectrum of P(PEGMA)-*b*-P(DMAEMA-*co*-CPLAMA) copolymer is represented in Fig. 1. In addition to P(PEGMA) signals, the new peaks related to the both P(CPLAMA) and P(DMAEMA) (with little difference on the ratio of the integral for the signals) were presented. The dominant CPLA resonances were observed at 5.20–5.17 ppm (CH) and at 1.61–1.59 ppm ranges (CH_3_) assigned to P(CPLAMA) blocks, and signals at 2.42, 2.75 and 4.29 ppm were characteristics of the methyl and methylene protons in DMAEMA units. The molecular composition of the copolymer was determined based on the ratios of the integral of the methoxy protons in PEG (s, *δ* 3.31) to the integral of the signal of the –CH_3_ protons in cholesterol group (k, *δ* 0.67) to the integral of the –CH_3_ protons of DMAEMA (a, *δ* 4.29) (Fig. 1). It was found that composition ratio of P(PEGMA):P(CPLAMA): P(DMAEMA) was resembled the feed ratio. The molar masses, average degree of polymerization (DP) and copolymer composition are listed in Table 1. GPC curve of copolymer exhibited a unimodal, single peak with the molecular distribution 1.86 (Table 1).

**Fig. 2:**
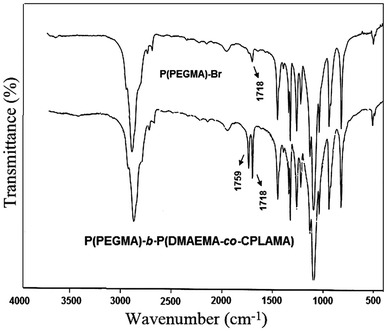
FT-IR spectra of P(PEGMA)-Br and P(PEGMA)-*b*-P(DMAEMA-*co*-CPLAMA)

Figure 2 shows the related FT-IR spectra of P(PEGMA)-Br and P(PEGMA)-*b*-P(DMAEMA-*co*-CPLAMA) copolymer. The characteristic absorption peaks were observed in the wave number region of C–H stretching modes at 2,888–2,949 cm^−1^ and the ester carbonyl groups at 1,717–1,758 cm^−1^. In addition, new absorption bands at 1,280 cm^−1^ due to the C–N stretching modes of amine groups were observed. The absence of a peak originating from C=C stretching in copolymer spectrum (about 1,620 cm^−1^) indicated successful copolymerization of monomers.

### Preparation and characterization of polymeric micelles

The co-solvent evaporation method was used to fabricate the copolymeric micelles. These amphiphilic copolymers could be assembled into micelles in aqueous solutions, and amphiphilic polymers could be assembled into the micelles in the aqueous solutions. It was reasonable that the hydrophobic CPLA was mainly in core of the micelles, whereas hydrophilic PEG side chains were in micelles shell. The system was cationic at low pH values (p*K*_a__PDMAEMA_ ~7.3–7.5 in water) due to the protonation of amine groups, but behaved as a hydrophobic weak polybase at high pH ranges.

#### pH-responsive behavior of the micelles

**Fig. 3:**
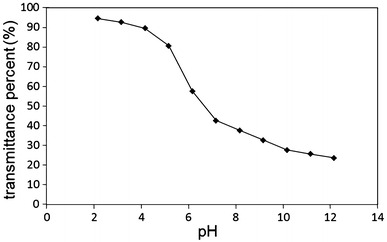
**a** pH dependence of transmittance at the wavelength of 500 nm for polymeric micelles in water at 25 °C (*c* = 2 mg mL^−1^)

**Fig. 4:**
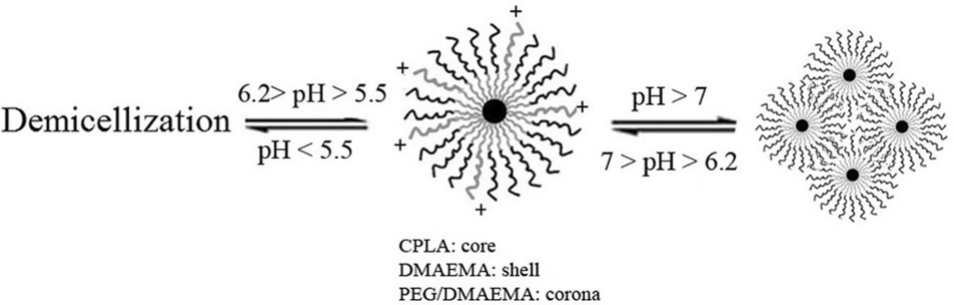
Schematic representation of the structural deformation of the P(PEGMA)-*b*-P(DMAEMA-*co*-CPLAMA) micelles with pH value

To evaluate the responsive ability, the polymeric micelles were treated with different pH values of the buffered solutions. Figure 3 shows the pH dependence of the UV–vis light transmittance on the copolymeric micelle solutions. The pH-dependent turbidity measurements should reflected the macroscopic changes of copolymeric micelles in water. By decreasing pH, the solution was transformed from opaque to transparent, indicating the obvious pH-responsive property for P(PEGMA)-*b*-P(DMAEMA-*co*-CPLAMA) copolymer. The pH transition of micelles was determined at pH 6.20 (Fig. 3). The copolymer was easily dissolved in aqueous solution at pH < 5.5 because the hydrophilicity of the polymer is relatively high due to the protonation of amine groups. The micelles aggregated rapidly above pH 7 to form micro particles due to increased lipophilic properties in copolymeric backbone. Micelles were immediately formed in the range of pH 5.5 (unimer region) to 6.5. The transition process was reversible by tuning the pH value (Fig. 4). The response to pH was very sharp and rapid.

#### Critical micelle concentration (CMC)

**Fig. 5:**
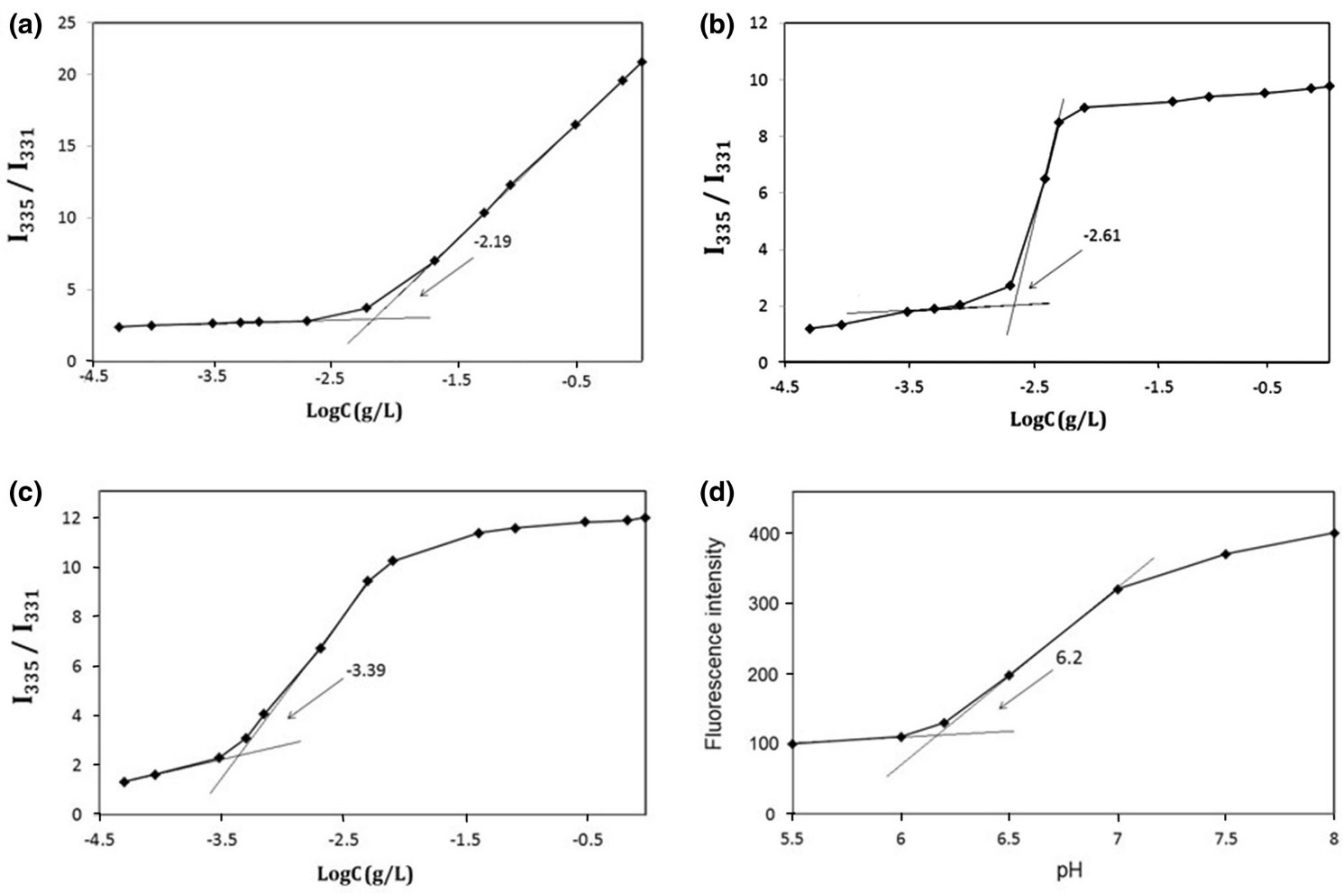
The intensity of *I*_335_/*I*_331_ ratio in pyrene fluorescence emission spectra as a function of log C of the copolymer at **a** pH 6, **b** pH 6.5, and **c** pH 7.0 at 25 °C (*c* = 5 × 10^−4^–1 mg mL^−1^), *λ*_ex_ = 335 nm, [pyrene] = 6 × 10^−7^ M; **d** the intensity of *I*_335_ nm in the emission spectra as a function of pH of the copolymer at 25 °C (*c* = 2 mg mL^−1^), *λ*_ex_ = 335 nm, [pyrene] = 6 × 10^−7^ M

The formation of micelles in aqueous phase at pH 6.0, 6.5 and 7.0 was confirmed by fluorescence technique using pyrene as a probe (Kabanov et al. 1995). Pyrene is a hydrophobic fluorescence probe that preferentially partitions into the hydrophobic core of the micelle. Consequently, its quantum yield increases due to the lowered environmental polarity, compared with the water environment. This provides a measure for both the presence of such regions and their hydrophobicities (Wang et al. 2007). The excitation of pyrene in the aqueous solutions containing various polymeric samples concentrations was achieved at 335 nm and emission spectra of pyrene were recorded. In all cases, the concentration of pyrene was kept at 6 × 10^−7^ M (the solubility of pyrene in water). Figure 5a–c shows plots of the pyrene fluorescence intensity ratio *I*_335_/*I*_331_, (*I*_335_, the first peak on the excitation spectra; *I*_331_, the third peak) versus the logarithm of the two samples concentration. The CMC values were determined from the intersection of the two tangent lines. The result is summarized in Table 2. The minimum value of the P(PEGMA)-*b*-P(DMAEMA-*co*-CPLAMA) polymer CMC was determined to be 2.45 mg L^−1^ at pH 6.2.

**Table 2:** Characterization of P(PEGMA)-*b*-P(DMAEMA-*co*-CPLAMA) micelles

P(PEGMA)-*b*-P(DMAEMA-*co*-CPLAMA)	Mean size (nm)^a^	Polydispersity index^a^	CMC (mg L^−1^)	Loading capacity (%)	Loading efficiency (%)
pH 6	–	–	6.46	–	–
pH 6.2	152 ± 13.9	0.15 ± 0.02	2.45	8	44
pH 7	3270 ± 267	4.34 ± 0.04	40.74	–	–

^a^Determined by dynamic light scattering (DLS)

The variation of pyrene fluorescence intensity at pH range 5.50–8.00 is shown in Fig. 5d. At pH < 5.5, fluorescence intensity values became very low due to the destruction of micelles. pH > 6.0 led to the abrupt increase in fluorescence intensity, reflecting the initiation of the formation of core–shell micelles. The inter-micellar association to form micellar aggregates occurred through further increasing pH (>7.0).

#### Morphology and size of micelles

**Fig. 6:**
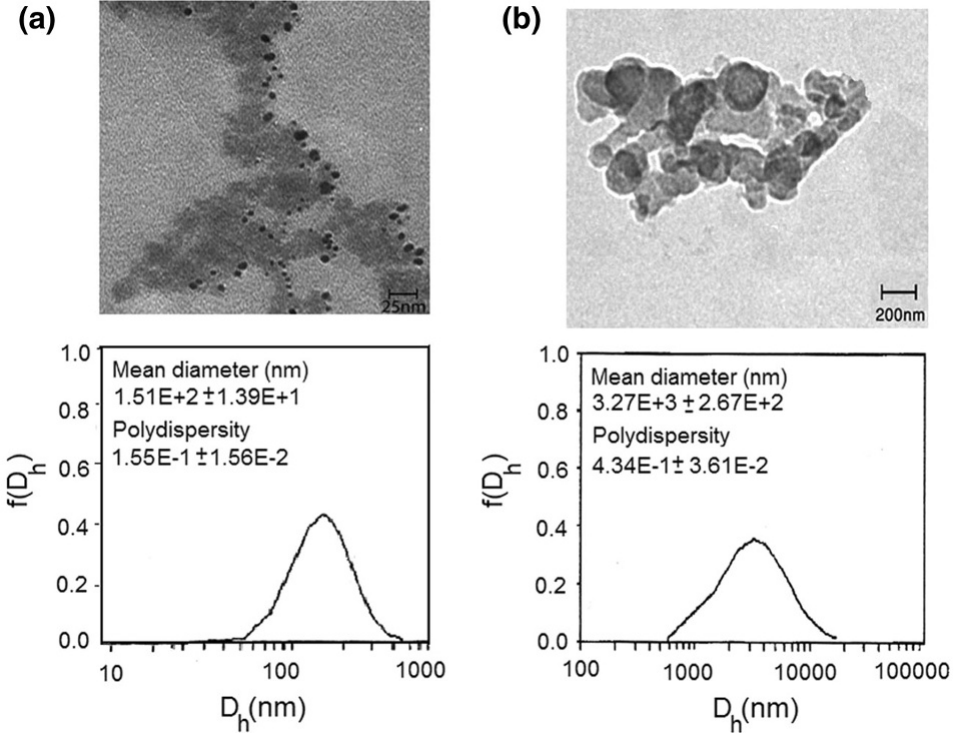
TEM images and size distributions of the **a** polymeric micelles at pH 6.2, **b** aggregated micelles from the polymer at pH 7.0 (polymer concentration of 2 mg mL^−1^)

The influence of aqueous solution pH on micellization behavior of copolymer was studied by DLS and TEM (Fig. 6). At acidic media, the highly protonated amine groups presented the hydrophilic character. As a result, the micellization was not attained due to the electrostatic repulsion forces between the ionic amine groups on the outer shells which kept polymer chains apart and prevented micellization. In the range of pH 5.5–6.2, micelles possessing cores were formed by cholesteryl moieties and partially protonated amine groups with PEG coronas (Fig. 4). The micelles began to shrink abruptly at around pH 6.2, which is due to the collapse of the PDMEMA chain the in shell of the micelles. Further increase in pH (pH > 7) led to increase in hydrodynamic diameter, which attributes to the aggregation of collapsed micelles (Fig. 6; Table 2). Moreover, pH effects on the diameter were a reversible procedure, and micelles could be recovered when pH was adjusted below 5.5 as shown in Fig. 4. Figure 6 shows TEM images of micelles prepared at pH 6.2 and 7. TEM pictures of copolymer micelles showed that the self-assembled micelles were well dispersed as individual micelles with spherical shapes at pH 6.2 (Fig. 6a). The diameters of 98.4 % corresponding micelles were ~152 nm. Polydispersity index (PDI) of micelles determined by DLS was low about 0.15 that also reinforced the successful micelle formation. At pH > 7, the totally neutralized amine groups led to form micellar aggregates possessing cholesteryl moieties and PDMAEMA in core and PEG in corona. TEM picture of micelles showed an irregular aggregation above transition pH 7.0 which appeared by the complete neutralization of amine content in basic condition (Fig. 6b). Micro particles resulted from micelles aggregation could also be confirmed by the results obtained from DLS (approximately >3 μm).

#### pH-dependent release of the naproxen from the micelles

Naproxen is a non-steroidal anti-inflammatory drug commonly used for the reduction of mild to moderate pain, fever, inflammation (Srinivas and Feldman 2009). Herein, naproxen was used as a model drug and loaded into the pH-responsive polymeric micelles. Naproxen-loaded micelles were prepared by the dialysis method of mixed solution of polymer and naproxen in THF against PBS with respective phase transition pH. It was found that wt% of the free drug naproxen was loaded into P(PEGMA)-*b*-P(DMAEMA-*co*-CPLAMA) micelles (*W*_total_ = 2 mg). The drug loading capacity and drug loading efficiency of polymeric micelles were 8 and 44 %, respectively (Table 2). Naproxen release behavior from polymeric micelles was studied in vitro by the dialysis method in the PBS solution (pH 2–12) at 37 °C.

**Fig. 7:**
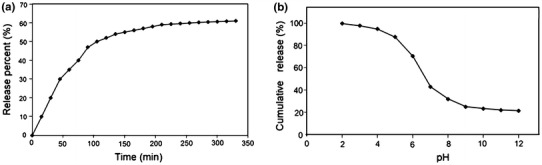
**a** In vitro release profiles of naproxen from polymeric micelles at 37 °C at pH 6.2. **b** Cumulative release of naproxen at different pH after 8 h. Data were presented as the mean ± standard deviation (*n* = 3)

Figure 6a presents in vitro release profiles of the naproxen-loaded micelles in PBS at transition pH 6.2. Naproxen was permeated at a relatively higher rate as 90 % of naproxen was transmitted in 8 h when the drug was used without micelles. However, during the same time period, ~50–60 % of naproxen released from polymeric micelles. As shown in Fig. 7, the release profiles show that naproxen was released quickly from micelles in the first stage and then the drug release could be sustained over a prolonged time at pH 6.2 (around the intestinal pH).

The naproxen release from copolymeric micelles was pH dependent, and the release rate at acidic pH was much faster than basic pH (Fig. 7b). At acidic pH, amine groups in the DMAEMA moieties are protonated. Therefore, fast drug release from micelles is observed due to the repulsion forces among the large amount of positively-charged amine groups which disrupted micelles shell. Obviously, naproxen release from micelles at basic pH was much slower than the release at acidic pH. It suggested that the encapsulated drugs could be sustained release in basic environment (Penga and Zhanga 2009). It can be mentioned that the pH differences within various tissues and cellular compartments are more subtle; for instance, the cancerous tissues have slightly acidic extra-cellular environment (pH 5–7.2). Therefore, the pH-dependent release behavior is of particular interest in achieving the drugs’ targeting through the micelles. As a result, P(PEGMA)-*b*-P(DMAEMA-*co*-CPLAMA) copolymeric micelles as the excellent pH-responsive drug nano-carriers may represent a highly promising approach to achieve the fast controlled drug release (Valerii et al. 2012).

## Conclusion

In this work, the synthesis and properties of a pH-sensitive copolymer were studied and its potential application as targeting drug carriers was also investigated. Amphiphilic P(PEGMA)-*b*-P(DMAEMA-*co*-CPLAMA) copolymer was synthesized by reaction between PEGMA and CPLAMA along with DMAEMA. The copolymer exhibited a phase transition pH around 6.2. The prepared copolymer was capable of self-assembling into nano-sized spherical micelles in aqueous solution with the diameter of around 152 nm determined by DLS. pH values were also affected the aggregation process. The micelle size could be adjusted through the alteration of solution pH values by increasing pH values. The release rate of drug from polymeric micelles was also decreased by increasing pH values. The problem of aggregation above pH 7 may be resolved with optimization of copolymer properties in modifying the copolymer structure like increasing the PEGMA block length. Therefore, these stealth polymeric micelles might find potential applications in intelligent drug delivery system for cancer theraphy after optimization.
